# Molecular markers of resistance to sulphadoxine-pyrimethamine one year after implementation of intermittent preventive treatment of malaria in infants in Mali

**DOI:** 10.1186/1475-2875-9-9

**Published:** 2010-01-10

**Authors:** Alassane Dicko, Issaka Sagara, Abdoulaye A Djimdé, Sidy O Touré, Mariam Traore, Souleymane Dama, Abdoulbaki I Diallo, Amadou Barry, Mohamed Dicko, Oumar M Coulibaly, Christophe Rogier, Alexandra de Sousa, Ogobara K Doumbo

**Affiliations:** 1 Malaria Research and Training Center, Department of Epidemiology of Parasitic Diseases, Faculty of Medicine Pharmacy and Dentistry, University of Bamako, PO Box 1805 Bamako, Mali; 2 Department of Public Health, Faculty of Medicine Pharmacy and Dentistry, University of Bamako, PO Box 1805 Bamako, Mali; 3 Centre de santé de référence de Kolokani, Région de Koulikoro, Mali; 4 Institut de Recherche Biomédicale des Armées IRBA - ex-IMTSSA & UMR6236-URMITE, Allée du Médecin colonel Jamot, Parc du Pharo, BP60109, 13262 Marseille cedex 07, France; 5 UNICEF-Head Quarters, 3 UN Plaza, New York, NY 10017, USA

## Abstract

**Background:**

Intermittent preventive treatment in infants (IPTi) with sulphadoxine-pyrimethamine (SP) given during routine vaccinations is efficacious in preventing malaria disease and shows no interaction with the vaccines. However, there is a fear that IPTi may result in a rapid increase of parasite resistance to SP.

**Methods:**

To evaluate the impact of IPTi on SP-resistance point mutations, the 22 health sub-districts in the district of Kolokani, Mali, were randomized in a 1:1 ratio and starting in December 2006, IPTi with SP was implemented in 11 health sub-districts (intervention zone), while the other 11 health sub-districts served as the control (non-intervention zone). Blood smears and blood dots on filter paper were obtained from children aged 0-5 years, randomly selected in each of heath sub-districts during two cross-sectional surveys. The first survey was conducted in May 2007 before the start of the transmission season to collect baseline prevalence of the molecular markers of resistance to SP and the second in December 2007 after the end of the transmission season and one year after implementation of IPTi. A total of 427 and 923 randomly selected blood samples from the first and second surveys respectively were analysed by PCR for *dhfr *and *dhps *mutations.

**Results:**

Each of the three *dhfr *mutations at codons 51, 59 and 108 was present in 35% and 57% of the samples during the two surveys with no significant differences between the two zones. *Dhps *mutations at codons 437 and 540 were present respectively in about 20% and 1% of the children during the two surveys in both zones at similar proportion. The prevalence of quadruple mutants (triple *dhfr*-mutants + *dhps*-437G) associated with *in-vivo *resistance to SP in Mali after one year implementation of IPTi was also similar between the two zones (11.6% versus 11.2%, p = 0.90) and to those obtained at baseline survey (10.3% versus 8.1%).

**Conclusion:**

This study shows no increase in the frequency of molecular markers of SP resistance in areas where IPTi with SP was implemented for one year.

## Background

Malaria remains a major cause of morbidity and mortality with an estimate of 881,000 deaths per year of which 91% occur in sub-Saharan Africa, mainly in infants and children under five years of age [1]. In the absence of a vaccine, simple and effective control strategies are urgently needed to reduce the malaria burden in sub-Saharan Africa. Several randomized controlled trials have demonstrated that Intermittent Preventive Treatment of malaria in infants (IPTi) with sulphadoxine-pyrimethamine (SP) given during routine vaccinations of the Expanded Programme of Immunization (EPI) at approximately two, three and nine months of age, result in a reduction of the incidence of clinical malaria by 22 to 59% [2-8], without showing interactions with EPI vaccines [2,4,9]. Although the efficacy and safety as well as the lack of interaction with EPI vaccines of IPTi in various endemic areas were well established [10-12], there have been concerns regarding the possibility that this strategy may cause an increase of *Plasmodium falciparum *resistance to SP, jeopardizing the use of SP not only for the prevention of malaria in infants, but also in pregnant women [13-15], an intervention widely adopted in many countries in sub-Saharan Africa. SP is known to rapidly select for mutant SP-resistant parasites [16-19], especially when given in prophylaxis, despite the reassuring predictions from mathematical modeling [13,20]. A report on the impact of IPTi with SP on the resistance of *P. falciparum *to SP in Mozambican children found no difference in the frequencies of *dhfr *and *dhps *mutations in children with a clinical malaria episode two months after the third dose of IPTi [21]. However, this study was conducted in the context of a placebo-controlled randomized trial with a limited number (n = 174) of samples. An evaluation in a context of large-scale implementation was needed to adequately address the impact of IPTi on SP drug resistance. This study evaluated the impact of IPTi on the selection SP-resistant *dhfr *and *dhps *mutations in *P. falciparum *after 12 months of implementation of IPTi in the district of Kolokani, Mali, where UNICEF pilot implementation of IPTi with SP was undertaken as part of a large operational research study in six African malaria endemic countries.

## Methods

### Study site

The study was conducted in the district of Kolokani, Mali. The district of Kolokani is an administrative subdivision of the region of Koulikoro. The town of Kolokani is located approximately 140 km north of Bamako. The district covers 14,380 km^2 ^and is divided into 22 health sub-districts. Each health sub-district is composed of several villages and has a health care center under the responsibility of a physician or chief-nurse and is staffed with at least two or three midwives and vaccinators. Three physicians ensure the coordination of health activities at the district level. The total population was 214,509 inhabitants. The children under one year of age represent about 4% of the total population. Malaria transmission is seasonal (July to November) with parasite prevalence in children 0-5 years of 45% at the end of the no-transmission season and above 70% at the end of the transmission season. First-line treatment of uncomplicated malaria was artesunate-amodiaquine or artemether-lumefantrine. Quinine was recommended for the treatment of severe malaria and SP was recommended prophylaxis for pregnant women.

### Study participants and design

The study was a cluster-randomized trial. Twenty-two health sub-districts were randomized into two groups in a 1:1 ratio and IPTi was implemented in 11 health sub-districts (intervention zone) while the other 11 health sub-districts served as the control (non-intervention zone). The implementation of IPTi started in December 2006 and consisted of the administration of ½ tablet of SP alongside the DTP2, DTP3 and Measles routine immunizations. Afridoxine^® ^(manufactured by Fourrts Laboratories Pvt. Ltd, Tamil Nadu, India), a breakable and dispersible form of SP was used after quality controls at the Laboratoire National de Santé in Bamako, Mali. Two cross-sectional surveys were performed. The first survey was performed in May 2007 a few months after the start of the IPTi-implementation, but before the start of the transmission season to collect baseline prevalence of the molecular markers of SP resistance. The second cross-sectional survey was conducted after one year of implementation in December 2007 after the end of the transmission season, to assess the impact of IPTi on the selection of SP-resistant *dhfr *and *dhps *mutations in *P. falciparum*. Children aged 0-5 years randomly selected in each of the health sub-districts were screened for malaria parasitaemia using thick smears obtained by finger pricking. Filter paper blood dots were also collected at the same time for the analysis of SP resistance molecular markers.

### Laboratory methods

Thick smears were Giemsa-stained and examined by microscopy. Parasite density was determined by counting parasites against 300 leucocytes using a conversion factor of 25, based on an assumed leukocyte count of 7,500/μl. Filter papers samples were air-dried and stored at room temperature in labeled individual envelopes. Preliminary genotyping of one hundred samples demonstrated that samples with a parasitaemia less than 500 parasites/μl did not consistently yield PCR product. Therefore, only filter paper samples from children with *P. falciparum *mono-infection parasitaemia greater or equal to 500 asexual stages per μl of blood were analysed by PCR for *dhfr *and *dhps *point mutations. Because of a higher than expected number *P. falciparum *mono-infection positive samples, 427 samples of the baseline survey and 923 samples of end of one year implementation survey were randomly selected and tested by PCR. Of the 427 baseline survey samples, 274 samples (150 in the intervention zone and 124 in the non-intervention zone) were successfully genotyped for all three *dhfr *and two *dhps *mutations, while the remaining were successfully tested for one, two, three or four mutations. Of the 923 randomly selected samples of end of one-year implementation survey, 742 samples (379 in the intervention zone and 363 in the non-intervention zone) were successfully genotyped for all three *dhfr *and two *dhps *mutations, and the remaining for one, two, three or four mutations.

A simple DNA extraction method for filter paper strips was used [22]. Briefly, approximately 1 × 2 mm piece of blood-soaked filter paper was placed in 50 μl of methanol for 15 min. The methanol was poured off and the paper heated at 95-100°C in 50 μl of water for 10 minutes. Five microliters of the resulting solution was used as PCR template for the first round of the Nested PCR. One microliter of the product of the first PCR was amplified in the second round of PCR. Samples that failed to yield PCR amplification with the methanol method were re-extracted using an alternative chelex-based method [23]. *Dhfr *and *dhps *genotypes were determined using nested mutation-specific PCR and/or PCR-RFLP according to published methods [16,23]. *Dhfr *mutations at codons 51, 59 and 108 and *dhps *mutations at codons 437 and 540 were analysed. One third of the PCR gels were examined by a senior scientist external to the study. The conformity of the gels with standard criteria for PCR gel validation and the fidelity of the transcription of lab notebook (source documents) with electronic files were checked. Error cases were corrected and PCR analyses repeated as needed.

### Study endpoints

The primary endpoint was the frequency of quadruple mutant of molecular markers (*dhfr *51, 59 and 108 and *dhps *437) after one year of IPTi implementation. Secondary endpoints were the frequencies of single mutations as well as the triple mutants (*dhfr *51 + 59 + 108) determined by PCR at the end of one year of implementation and frequencies of these individual and multiples *dhfr *and *dhps *mutations at baseline. The frequency of a particular mutant was calculated as the proportion of the specific mutant samples among the total number of samples successfully analysed for this mutation. Similarly, the frequencies of triple, quadruple and quintuple mutants were determined as the proportion of subjects with the three, four and five mutations among the total numbers of samples tested for the each.

### Sample size

Assuming a proportion of the primary endpoint (quadruple mutants, *dhfr *51, 59 and 108 and *dhps *437) of 8% in control zone and 14% in IPTi intervention zone, one side p-value 0.025, a power of 80%, and inter class correlation using 0.01, a sample size of 380 children with positive parasitaemia in each group with a minimum of 22 subjects and average of 35 subjects per cluster were needed. Assuming a prevalence of *P. falciparum *parasitaemia of 35% and about 10% lost or unsuccessful samples, about 110 subjects were need to be screened per cluster resulting in a total of 2,420 children for the two arms.

### Statistical analysis

Cases of mixed infection (wild type and mutant) were categorized as mutant throughout the analysis. Prevalence of single mutations as well as the triple mutant (*dhfr *51 + 59 + 108) and quadruple mutant (triple mutant + *dhps *437) genotypes were determined and compared between the IPTi implementation and control zones with 95% confidence intervals around the odds ratios adjusted for non-independence within health sub-districts using cluster option in Stata (version 9, Houston Texas USA). Proportions of categorical variables were compared using Pearson's Chi square test. Student t test was used for the comparison of mean ages and Mann-Whitney test for the comparison of parasite densities between the two zones.

### Ethical considerations

Ethical clearance was obtained from the Ethical Committee of the Faculty of Medicine Pharmacy and Dentistry of the University of Bamako, Mali, and written informed consent from a parent or legal guardian was obtained prior to the enrollment of the child in the survey.

## Results

### Prevalence of *dhfr *and *dhps *mutations at baseline survey

A total of 2,437 subjects were screened (1,213 in the intervention zone and 1,224 in the non-intervention zone). Among these, 1,157 subjects (573 in the intervention zone and 584 in the non-intervention zone) had *P. falciparum *parasitaemia and 858 (429 in each zone) with parasitaemia equal or above 500/μl were selected. Characteristics of subjects enrolled are presented in Table 1. There were no significant differences between the two groups in terms of age, parasite prevalence, parasite density, and prevalence of parasitaemia equal or above 500/μl.

**Table 1:** Characteristics of subjects enrolled in the IPTi intervention and non-intervention zone in Kolokani in May 2007.

	No IPTi	IPTi	p
Number screened	1224	1213	

Mean age (months)	34.1	32.9	0.15

Gender (Male) (%)	53.4	50.9	0.21

Parasite prevalence (%)	47.7	47.2	0.82

Median parasitaemia (per μl)	950	1275	0.44

Prevalence of parasitaemia ≥ 500/μl (%)	35.1	35.2	0.87

Prevalence of individual mutations, triple and quadruple mutations in intervention and non-intervention zones are presented in Table 2. Prevalence of individual *dhfr *and *dhps *mutations and triple *dhfr *mutation were similar between the intervention and non-intervention zones. The quadruple mutation was present in 9.2% of all the samples with similar frequency in the two zones (10.3% in the intervention zone and 8.1% in the non-intervention zone).

**Table 2:** Prevalence of molecular markers associated with *P. falciparum *resistance to SP in IPTi intervention and non-intervention zone in Kolokani at baseline in May 2007.

	No IPTi		IPTi		p
**Molecular Markers**	**n**	**Prevalence**	**n**	**Prevalence**	

DHPS 437	333	20.1%	315	17.5%	0.39

DHPS 540	265	0.4%	313	1.6%	0.22

DHFR 51	209	35.4%	216	38.4%	0.52

DHFR 59	220	48.2%	206	47.6%	0.90

DHFR 108	223	43.0%	242	38.0%	0.27

Triple DHFR mutation	179	34.6%	184	35.8%	0.81

Quadruple mutation	161	8.1%	174	10.3%	0.47

### Prevalence of *dhfr *and *dhps *mutations after one year of IPTi implementation

A total of 2,453 subjects were screened (1,227 in the intervention zone and 1,226 in the non-intervention zone). Among these, 1,693 (855 in the intervention zone and 838 in the non-intervention zone) had *P. falciparum *parasitaemia and 1,490 (735 in the intervention zone and 755 in the non-intervention zone) had *P. falciparum *parasitaemia equal or above 500/μl. Characteristics of children sampled in the two zones are presented in Table 3. There were no significant differences between the two groups in terms of age, parasite prevalence, parasite density and prevalence of parasitaemia equal or above 500/μl.

**Table 3:** Characteristics of subjects enrolled in the IPTi intervention and non-intervention zone in Kolokani in December 2007 after one year of implementation.

	No IPTi	IPTi	p
Number screened	1226	1227	

Mean age (months)	32.9	33.0	0.96

Gender (Male) (%)	51.3	50.3	0.63

Parasite prevalence (%)	71.5	73.3	0.32

Median parasitaemia (per μl)	3325	3850	0.06

Prevalence of parasitaemia ≥ 500/μl (%)	62.7	64.8	0.31

Prevalence of individual *dhfr *and *dhps *mutations as well as multiple mutations (*dhfr *triple mutation and quadruple mutation (triple +*dhps *437)) are presented in Table 4. There were no significant differences in the frequency of individual mutations between intervention and non-intervention zones. About half of the children harboured one of three *dhfr *mutations in both the intervention and non-intervention zones with a frequency of the triple *dhfr *mutation of 38.5% in the intervention zone compared to 44.8% in the control zone. About 20% of the children with parasitaemia in each zone were positive for parasites with the mutation 437 on *dhps *while only about 1% harboured parasites with the *dhps *540 mutation. Similar patterns were found when the analysis was done by age categories for 2-24 months and 25-60 months.

**Table 4:** Prevalence of molecular markers associated with *P. falciparum *resistance to SP in IPTi intervention and non-intervention zone in Kolokani in December 2007 after one year of implementation.

	No IPTi		IPTi		OR	95% CI	p
**Molecular Markers**	**n**	**Prevalence** **(95% CI)**	**n**	**Prevalence** **(95% CI)**			

DHPS 437	432	19.4% (15.1 - 24.6)	479	21.7% (17.4-26.7)	1.15	0.59- 2.24	0.68

DHPS 540	428	0.9% (0.2-3.1)	474	1.0% (0.3-3.1)	1.13	0.16-7.99	0.90

DHFR 51	379	56.5% (50.1-62.6)	402	51.0% (44.9-57.1)	0.80	0.52-1.23	0.32

DHFR 59	376	55.3% (48.9-61.5)	402	51.0% (44.9-57.1)	0.84	0.50-1.40	0.51

DHFR 108	375	56.8% (50.4-63.0)	392	47.7% (41.5-53.9)	0.69	0.47-1.03	0.07

Triple DHFR mutation (DHFR 51, 59 and 108)	368	44.8% (38.5-51.3)	384	38.5% (32.6-44.8)	0.77	0.50-1.19	0.24

Quadruple mutation (DHFR 51, 59 and 108 + DHPS 437)	367	11.2% (7.6-16.0)	379	11.6% (8.1-16.4)	1.04	0.51-2.12	0.90

The prevalence of the quadruple mutants was also similar between the two zones: 11.6% in the intervention zone versus 11.2% in the control zone, odd ratio = 1.04 (95% CI 0.51-2.12) p = 0.90 (Table 4). When the data were analysed by age categories, similar frequencies of quadruple mutants were found in the two zones in children aged 2-24 months; 9.9% (14/142) in the intervention group versus 8.2% (12/147) in the control group (p = 0.62). The corresponding figures in children aged 25-60 months were 12.9% (29/236) in the intervention group and 13.2% (29/219) in the control group (p = 0.76).

Three of the overall 742 samples (0.8%) successfully genotyped for all three *dhfr *and two *dhps*, harboured quintuple mutants; all three in the non-intervention zone, with one in the younger age group (less or equal to 24 months) and the other two in the older age group (25-60 months).

## Discussion

This study showed no difference between intervention and non-intervention zones in the frequency of individual mutants in the *dhfr *and *dhps *genes, as well as in the triple *dhfr *and quadruple *dhfr dhps *mutants, suggesting that the implementation of IPTi with SP for one year does not result in an increase in the frequency of these molecular markers at the community level. This is consistent with the predictions using mathematical modelling that have indicated that IPTi is unlikely to appreciably shorten the useful life of the drug used [20], or to significantly impact on the spread of highly resistant parasites in areas where partial resistance is already established [13] as it is the case in Mali and in many countries in sub-Saharan Africa. A small scale placebo-controlled randomized trial of IPTi with SP in Mozambican children [21] found no difference in the frequency of *dhfr *and *dhps *mutations during the episodes of clinical malaria that occurred two months after the last dose of IPTi. Another study [24] found a higher frequency of infections exhibiting four mutations in *dhfr *and *dhps *in children of nine months of age, after a single dose of SP compared to those in the control group, but no difference in the rate of infections with isolates with less than four mutations between the two groups. Other small scale placebo-control randomized studies of IPT in children (IPTc) during a highly seasonal malaria transmission season did not find differences in the frequency of *dhfr *and *dhps *mutations [25] or *in vivo *response to SP [26] between those who received IPT with SP versus those who did not. Similar frequency of the *dhfr *triple mutant among antennal care attendees of early gestation who had not received IPT and delivering women who had received IPT with SP in pregnancy (IPTp) was reported in a study in Ghana [18]. As estimated in mathematical models, the drug pressure resulting from IPTi may be relatively small [20] and there is no evidence that IPT will lead to increased resistance to SP that could jeopardize the use of the strategy in many countries in sub-Saharan Africa. However, this study has assessed the impact of IPTi on molecular markers of SP resistance after only one year of implementation, and continuous monitoring of resistance to SP (both molecular and *in vivo*) would be warranted once IPT is to be implemented as policy for longer periods of time. Possible limitations of this study include i) potential bias due to the difference in the spread of resistance between this study target population (children between 0-5 years of age) and the IPTi target population (children less than one year of age), however the sub-groups analysis did not shown any difference between the two zones in the children 2-24 months at the time of the second cross-sectional survey, targeted by the intervention and ii) the possibility of cross-contamination between intervention and non-intervention zones. However, the use of IPTi in non-intervention zones is unlikely to bias our results as only a very small proportion (less than 1%) of children 0-23 months had received IPTi in the non-intervention zone, and a high coverage of IPTi (>90% based on health records) was achieved in the intervention zone. Furthermore, the frequency of quadruple mutants at baseline was 10.3% compared to 11.6% at the end of first year of intervention and 8.1% compared to 11.2% in the control group. Rapid selection under drug pressure of pre-existent resistant strains at the community level has been reported [27]. However, in the absence of structured *P. falciparum *populations, the flow of parasites between areas that are very close could have diluted *P. falciparum *populations that were selected under SP pressure in the intervention zones by unselected *P. falciparum *populations coming from non-intervention zones [28]. The use of threshold parasitaemia equal or above 500/μl is unlikely to bias our results since the prevalence of parasitaemia above this threshold was similar between the two zones, and it is unlikely that mutants parasite were more prevalent below this threshold in one zone compared to other.

In line with other studies in Mali and countries in West Africa [29-32], this study shows relatively high levels of individual *dhfr *mutations in the study area with half of the parasitemic children harbouring mutant genotypes and a prevalence of triple mutations of 44.5%. This may reflect the long history of usage of sulpha drugs including SP across the countries. Although the *dhfr *triple mutation was prevalent in 34.6 to 44.8% of infections, it is well established that this genotype does not correlate with *in vivo *SP failure in West Africa [31-33]. The quadruple mutation genotype most associated with *in vivo *SP failure in Mali (our unpublished data) and in Ghana [33] was similar between the two zones and present in about 11% of infections in overall at the end of one year of implementation. This is also consistent with rates for this genotype found by us in various parts of Mali and by others in the West African sub-region [31,34,35]. Similar to other reports from the sub-region, the *dhps *540E was very rare in this study [31,33]. However, here are reported the first cases of *dhps *540E found in Mali.

In conclusion, this study shows low prevalence of quadruple mutants (triple *dhfr *mutants + *dhps *437G) associated with *in vivo *resistance to SP in Mali and no evidence of increased frequency of molecular markers of SP resistance in areas where IPTi was implemented for one year.

## Competing interests

The authors declare that they have no competing interests.

## Authors' contributions

AD was the principal investigator of the study. He contributed to the study design, oversaw the study conduct, the data collection and contributed to the data analysis and interpretation. IS contributed to the study design and conducted the data collection. AAD contributed to the study design and oversaw the molecular analysis of the samples and interpretation of the data. Data collection in the field was done by ST, MT, SD, AID, AB, MD. OMC contributed to the study conduct and data collection. CR contributed to the study design and data analysis. AS contributed to the study design. OKD contributed to the design of the study and overseeing the data collection, analysis and interpretation. The manuscript was drafted by AD and all the authors contributed to revision and approved the final version.
